# *N*,*N*,*N*′,*N*′-Tetra­kis(2-hy­droxy-5-methyl­benz­yl)ethane-1,2-diamine dimethyl­formamide disolvate

**DOI:** 10.1107/S1600536811017934

**Published:** 2011-05-20

**Authors:** Nuan-Sheng Wang, Yong-Tao Wang, Xiu-Kai Guo, Tian-Duo Li

**Affiliations:** a Shandong Provincial Key Laboratory of Fine Chemicals, Shandong Polytechnic University, Jinan 250353, People’s Republic of China; b School of Petrochemical Engineering, Changzhou University, Changzhou 213164, People’s Republic of China

## Abstract

The title compound, C_34_H_40_N_2_O_4_·2C_3_H_7_NO, was synthesized by the Mannich condensation of ethane­diamine, formaldehyde and *p*-cresol. In the crystal, the tetra­phenol mol­ecule is arranged around an inversion center. The mol­ecule and the dimethyl­formamide solvate are linked through an O—H⋯O hydrogen bond. An intra­molecular O—H⋯N hydrogen bond occurs in the tetra­phenol mol­ecule, which may influence the mol­ecular confomation. Futhermore, C—H⋯O and π–π stacking inter­actions [centroid–centroid distance = 3.7081 (14) Å] stabilize the crystal packing, building a three-dimensional network.

## Related literature

For applications of the title compound, see: Liu *et al.* (2007 ▶); Tshuva *et al.* (2000 ▶); For related structures, see: Hou *et al.* (2010 ▶); Higham *et al.* (2006 ▶); Farrell *et al.* (2007 ▶).
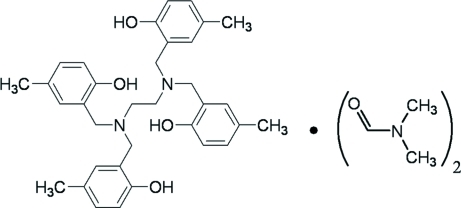


## Experimental

### 

#### Crystal data

C_34_H_40_N_2_O_4_·2C_3_H_7_NO
*M* *_r_* = 686.87
Monoclinic, 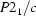

*a* = 11.574 (2) Å
*b* = 6.3557 (12) Å
*c* = 26.343 (5) Å
β = 94.939 (3)°
*V* = 1930.7 (6) Å^3^
*Z* = 2
Mo *K*α radiation
μ = 0.08 mm^−1^
*T* = 298 K
0.50 × 0.32 × 0.27 mm

#### Data collection

Bruker SMART APEX diffractometer
9607 measured reflections
3569 independent reflections
2667 reflections with *I* > 2σ(*I*)
*R*_int_ = 0.029

#### Refinement

*R*[*F* ^2^ > 2σ(*F* ^2^)] = 0.058
*wR*(*F* ^2^) = 0.154
*S* = 1.07
3569 reflections
232 parameters
H-atom parameters constrained
Δρ_max_ = 0.19 e Å^−3^
Δρ_min_ = −0.15 e Å^−3^

### 

Data collection: *SMART* (Bruker, 2000 ▶); cell refinement: *SAINT* (Bruker, 2000 ▶); data reduction: *SAINT*; program(s) used to solve structure: *SHELXS97* (Sheldrick, 2008 ▶); program(s) used to refine structure: *SHELXL97* (Sheldrick, 2008 ▶); molecular graphics: *ORTEPIII* (Burnett & Johnson, 1996 ▶), *ORTEP-3 for Windows* (Farrugia, 1997 ▶) and *PLATON* (Spek, 2009 ▶); software used to prepare material for publication: *SHELXTL* (Sheldrick, 2008 ▶).

## Supplementary Material

Crystal structure: contains datablocks I, global. DOI: 10.1107/S1600536811017934/dn2686sup1.cif

Structure factors: contains datablocks I. DOI: 10.1107/S1600536811017934/dn2686Isup2.hkl

Supplementary material file. DOI: 10.1107/S1600536811017934/dn2686Isup3.cdx

Supplementary material file. DOI: 10.1107/S1600536811017934/dn2686Isup4.cml

Additional supplementary materials:  crystallographic information; 3D view; checkCIF report

## Figures and Tables

**Table 1:** Hydrogen-bond geometry (Å, °)

*D*—H⋯*A*	*D*—H	H⋯*A*	*D*⋯*A*	*D*—H⋯*A*
O1—H1⋯N1	0.82	1.98	2.705 (2)	147
O2—H2⋯O3	0.82	1.87	2.690 (2)	177
C18—H18⋯O3^i^	0.93	2.56	3.368 (3)	145

Symmetry code: (i) 
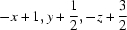
.

**Fig. 1.**
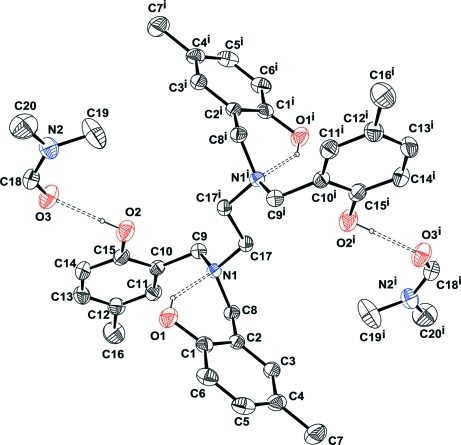
Molecular structure of the title compound with the atom labeling scheme. Displacement ellipsoids are drawn at the 30% probability level. H atoms are represented as small sphere of arbitrary radii. Hydrogen bonds are shown as dashed lines. Hydrogen atoms not involved in hydrogen bonding have been omitted for clarity. [Symmetry code: (i) -x+1, -y+1, -z+1]

**Fig. 2.**
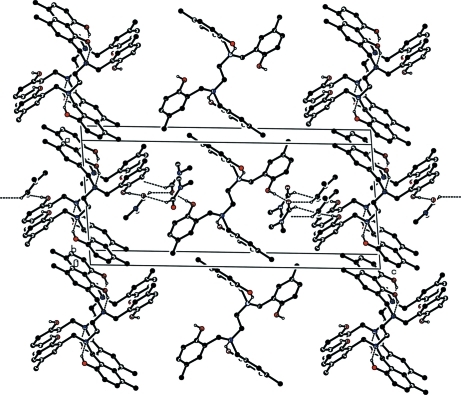
Molecular packing of the title compound viewing along the crystallographic b-axis. Hydrogen bonds are shown as dashed lines. Hydrogen atoms not involved in hydrogen bonding have been omitted for clarity.

## supplementary crystallographic information

### Comment

Multidentate aminophenol are of interest as metallochelators and as ligands for bioinorganic modeling, catalysis, and medical imaging.(Higham *et al.*, 2006; Farrell *et al.*, 2007). Some of them in combination with metals are used as active catalysts for alkenes polymerization (Tshuva *et al.*, 2000) and initiators in the ring-openingpolymerization of lactones (Liu *et al.*, 2007). Herein, we report the crystal structure of the title compound, 'C_34_H_40_N_2_O_4_.(C_3_H_7_NO)_2_'.

The *N*, *N*'-Tetrakis(2-hydroxy-5-methylbenzyl)-1, 2-ethanediamine molecule is arranged around inversion center located in the middle of the CH_2_-CH_2_ bond. The DMF solvate is linked to this molecule through O-H···O hydrogen bonds (Fig. 1). There is also a weak intramolecular O-H···N interactions which might influence the conformation of the molecule (Table 1) (Hou *et al.*, 2010).

The occurence of weak C-H···O interactions (Table 1) and π-π stacking between the symmetry related C1—C6 phenyl rings (Centroid to centroid distance of 3.7081 (14)Å, interplanar distance of 3.6891 (8)° and slippage of 0.375Å) result in the formation of a three dimensional network (Fig. 2)

### Experimental

The title compound was prepared by mixing ethylenediamine (1.0 mmol), paraformaldehyde (4.0 mmol) and *p*-cresol (10 mmol) were heated to 90°C and stirred for 18 h. This reaction requires no solvent nor inert atmosphere. At the end of the reaction, 10ml of ethanol was added to the mixtures to remove the unreacted *p*-cresol, then sonicated 10 minutes. Finally a white precipitate product was collected by filtration in 56% yield.

### Refinement

All H atoms were placed in idealized positions and treated as riding, with C—H = 0.93 Å (CH), 0.97 Å (CH_2_), 0.96 Å (CH_3_), O—H = 0.82 Å and and *U*_iso_(H) = 1.2 *U*_eq_(CH and CH_2_), *U*_iso_(H) = 1.5 *U*eq(CH_3_ and OH).

### Crystal data

**Table:**

C_34_H_40_N_2_O_4_·2C_3_H_7_NO	*F*(000) = 740
*M**_r_* = 686.87	*D*_x_ = 1.182 Mg m^−^^3^
Monoclinic, *P*2_1_/*c*	Mo *K*α radiation, λ = 0.71073 Å
Hall symbol: -P 2ybc	Cell parameters from 2283 reflections
*a* = 11.574 (2) Å	θ = 2.3–22.4°
*b* = 6.3557 (12) Å	µ = 0.08 mm^−^^1^
*c* = 26.343 (5) Å	*T* = 298 K
β = 94.939 (3)°	Block, colourless
*V* = 1930.7 (6) Å^3^	0.50 × 0.32 × 0.27 mm
*Z* = 2	

### Data collection

**Table:**

Bruker SMART APEX diffractometer	2667 reflections with *I* > 2σ(*I*)
Radiation source: fine-focus sealed tube	*R*_int_ = 0.029
graphite	θ_max_ = 25.5°, θ_min_ = 1.6°
φ and ω scans	*h* = −13→13
9607 measured reflections	*k* = 0→7
3569 independent reflections	*l* = 0→31

### Refinement

**Table:**

Refinement on *F*^2^	Primary atom site location: structure-invariant direct methods
Least-squares matrix: full	Secondary atom site location: difference Fourier map
*R*[*F*^2^ > 2σ(*F*^2^)] = 0.058	Hydrogen site location: inferred from neighbouring sites
*wR*(*F*^2^) = 0.154	H-atom parameters constrained
*S* = 1.07	*w* = 1/[σ^2^(*F*_o_^2^) + (0.0747*P*)^2^ + 0.2941*P*] where *P* = (*F*_o_^2^ + 2*F*_c_^2^)/3
3569 reflections	(Δ/σ)_max_ = 0.003
232 parameters	Δρ_max_ = 0.19 e Å^−^^3^
0 restraints	Δρ_min_ = −0.15 e Å^−^^3^

### Special details

**Table:**

Geometry. All esds (except the esd in the dihedral angle between two l.s. planes) are estimated using the full covariance matrix. The cell esds are taken into account individually in the estimation of esds in distances, angles and torsion angles; correlations between esds in cell parameters are only used when they are defined by crystal symmetry. An approximate (isotropic) treatment of cell esds is used for estimating esds involving l.s. planes.
Refinement. Refinement of *F*^2^ against ALL reflections. The weighted *R*-factor *wR* and goodness of fit *S* are based on *F*^2^, conventional *R*-factors *R* are based on *F*, with *F* set to zero for negative *F*^2^. The threshold expression of *F*^2^ > σ(*F*^2^) is used only for calculating *R*-factors(gt) *etc*. and is not relevant to the choice of reflections for refinement. *R*-factors based on *F*^2^ are statistically about twice as large as those based on *F*, and *R*- factors based on ALL data will be even larger.

### Fractional atomic coordinates and isotropic or equivalent isotropic displacement parameters (Å^2^)

**Table:**

	*x*	*y*	*z*	*U*_iso_*/*U*_eq_	
C1	0.81761 (16)	0.6724 (3)	0.50272 (8)	0.0459 (5)	
C2	0.80450 (15)	0.4662 (3)	0.48545 (7)	0.0399 (5)	
C3	0.86027 (16)	0.4078 (3)	0.44304 (7)	0.0472 (5)	
H3	0.8508	0.2709	0.4309	0.057*	
C4	0.92929 (17)	0.5440 (4)	0.41797 (8)	0.0546 (6)	
C5	0.94148 (18)	0.7469 (4)	0.43666 (9)	0.0602 (6)	
H5	0.9880	0.8417	0.4209	0.072*	
C6	0.88609 (17)	0.8110 (4)	0.47813 (9)	0.0567 (6)	
H6	0.8948	0.9487	0.4897	0.068*	
C7	0.9908 (2)	0.4688 (5)	0.37314 (10)	0.0846 (9)	
H7A	1.0649	0.4111	0.3851	0.127*	
H7B	1.0018	0.5849	0.3508	0.127*	
H7C	0.9450	0.3624	0.3551	0.127*	
C8	0.74176 (15)	0.3055 (3)	0.51447 (7)	0.0434 (5)	
H8A	0.7213	0.1869	0.4923	0.052*	
H8B	0.7933	0.2546	0.5428	0.052*	
C9	0.59865 (17)	0.2520 (3)	0.57452 (7)	0.0465 (5)	
H9A	0.6064	0.1064	0.5642	0.056*	
H9B	0.5174	0.2778	0.5787	0.056*	
C10	0.66842 (16)	0.2860 (3)	0.62476 (7)	0.0444 (5)	
C11	0.74762 (17)	0.1400 (4)	0.64530 (8)	0.0503 (5)	
H11	0.7593	0.0176	0.6271	0.060*	
C12	0.81018 (19)	0.1691 (4)	0.69194 (8)	0.0571 (6)	
C13	0.7899 (2)	0.3509 (5)	0.71814 (8)	0.0648 (7)	
H13	0.8299	0.3731	0.7498	0.078*	
C14	0.71194 (19)	0.5012 (4)	0.69888 (8)	0.0615 (6)	
H14	0.7001	0.6229	0.7174	0.074*	
C15	0.65167 (18)	0.4696 (4)	0.65193 (8)	0.0521 (6)	
C16	0.8989 (2)	0.0118 (5)	0.71276 (11)	0.0892 (9)	
H16A	0.9725	0.0439	0.7004	0.134*	
H16B	0.8751	−0.1269	0.7019	0.134*	
H16C	0.9057	0.0181	0.7493	0.134*	
C17	0.54400 (16)	0.4187 (3)	0.49300 (7)	0.0463 (5)	
H17A	0.5051	0.2856	0.4858	0.056*	
H17B	0.5782	0.4636	0.4624	0.056*	
N1	0.63564 (12)	0.3898 (3)	0.53418 (5)	0.0406 (4)	
O1	0.76581 (13)	0.7432 (3)	0.54411 (6)	0.0640 (5)	
H1	0.7179	0.6567	0.5520	0.096*	
O2	0.57293 (15)	0.6113 (3)	0.63071 (6)	0.0721 (5)	
H2	0.5563	0.6957	0.6525	0.108*	
C18	0.4736 (2)	1.0737 (4)	0.69918 (8)	0.0546 (6)	
H18	0.5089	1.1710	0.7219	0.066*	
C19	0.3253 (3)	0.9941 (6)	0.63369 (15)	0.1233 (13)	
H19A	0.3785	0.8864	0.6252	0.185*	
H19B	0.2982	1.0695	0.6034	0.185*	
H19C	0.2606	0.9307	0.6484	0.185*	
C20	0.3383 (3)	1.3486 (5)	0.67385 (12)	0.0963 (10)	
H20A	0.3860	1.4260	0.6990	0.144*	
H20B	0.2604	1.3422	0.6837	0.144*	
H20C	0.3385	1.4176	0.6414	0.144*	
N2	0.38342 (17)	1.1383 (3)	0.67004 (7)	0.0636 (5)	
O3	0.51586 (16)	0.8979 (3)	0.69941 (6)	0.0731 (5)	

### Atomic displacement parameters (Å^2^)

**Table:**

	*U*^11^	*U*^22^	*U*^33^	*U*^12^	*U*^13^	*U*^23^
C1	0.0384 (10)	0.0481 (13)	0.0502 (12)	0.0064 (9)	−0.0022 (9)	−0.0022 (10)
C2	0.0319 (9)	0.0477 (12)	0.0389 (10)	0.0071 (8)	−0.0024 (8)	0.0010 (9)
C3	0.0417 (11)	0.0552 (13)	0.0442 (11)	0.0071 (9)	0.0001 (9)	−0.0009 (10)
C4	0.0419 (11)	0.0752 (17)	0.0469 (12)	0.0068 (11)	0.0041 (9)	0.0115 (11)
C5	0.0422 (12)	0.0656 (17)	0.0723 (15)	−0.0005 (11)	0.0026 (11)	0.0213 (13)
C6	0.0442 (12)	0.0465 (13)	0.0783 (16)	0.0004 (10)	−0.0007 (11)	0.0036 (11)
C7	0.0739 (17)	0.120 (3)	0.0630 (15)	−0.0014 (17)	0.0253 (14)	0.0050 (16)
C8	0.0400 (10)	0.0458 (12)	0.0440 (11)	0.0088 (9)	0.0025 (8)	−0.0009 (9)
C9	0.0428 (11)	0.0514 (13)	0.0455 (11)	−0.0021 (9)	0.0049 (9)	−0.0026 (9)
C10	0.0452 (11)	0.0516 (13)	0.0377 (10)	−0.0029 (9)	0.0106 (8)	0.0003 (9)
C11	0.0540 (12)	0.0500 (13)	0.0477 (11)	−0.0015 (10)	0.0098 (10)	0.0024 (10)
C12	0.0546 (13)	0.0705 (16)	0.0458 (12)	−0.0007 (11)	0.0019 (10)	0.0075 (11)
C13	0.0586 (14)	0.093 (2)	0.0422 (12)	−0.0093 (13)	−0.0011 (11)	−0.0027 (13)
C14	0.0633 (14)	0.0755 (17)	0.0469 (12)	−0.0021 (12)	0.0109 (11)	−0.0160 (11)
C15	0.0515 (12)	0.0600 (15)	0.0458 (12)	0.0057 (10)	0.0107 (10)	−0.0030 (10)
C16	0.0865 (19)	0.100 (2)	0.0772 (18)	0.0155 (17)	−0.0128 (16)	0.0156 (16)
C17	0.0416 (10)	0.0563 (13)	0.0402 (10)	0.0066 (9)	−0.0011 (8)	−0.0110 (9)
N1	0.0364 (8)	0.0495 (10)	0.0361 (8)	0.0050 (7)	0.0033 (7)	−0.0033 (7)
O1	0.0680 (11)	0.0568 (11)	0.0690 (10)	−0.0011 (8)	0.0163 (8)	−0.0191 (8)
O2	0.0804 (12)	0.0741 (13)	0.0611 (10)	0.0264 (9)	0.0021 (9)	−0.0153 (9)
C18	0.0682 (14)	0.0544 (15)	0.0419 (11)	0.0012 (12)	0.0088 (11)	−0.0042 (10)
C19	0.123 (3)	0.123 (3)	0.114 (3)	−0.020 (2)	−0.048 (2)	−0.016 (2)
C20	0.096 (2)	0.089 (2)	0.103 (2)	0.0314 (18)	0.0033 (18)	0.0172 (18)
N2	0.0689 (12)	0.0650 (14)	0.0549 (11)	−0.0008 (10)	−0.0066 (10)	0.0031 (10)
O3	0.0999 (13)	0.0595 (11)	0.0612 (10)	0.0219 (10)	0.0149 (9)	−0.0026 (8)

### Geometric parameters (Å, °)

**Table:**

C1—O1	1.365 (2)	C12—C16	1.502 (4)
C1—C6	1.383 (3)	C13—C14	1.380 (3)
C1—C2	1.391 (3)	C13—H13	0.9300
C2—C3	1.388 (3)	C14—C15	1.381 (3)
C2—C8	1.500 (3)	C14—H14	0.9300
C3—C4	1.384 (3)	C15—O2	1.366 (3)
C3—H3	0.9300	C16—H16A	0.9600
C4—C5	1.383 (3)	C16—H16B	0.9600
C4—C7	1.508 (3)	C16—H16C	0.9600
C5—C6	1.375 (3)	C17—N1	1.462 (2)
C5—H5	0.9300	C17—C17^i^	1.518 (4)
C6—H6	0.9300	C17—H17A	0.9700
C7—H7A	0.9600	C17—H17B	0.9700
C7—H7B	0.9600	O1—H1	0.8200
C7—H7C	0.9600	O2—H2	0.8200
C8—N1	1.475 (2)	C18—O3	1.219 (3)
C8—H8A	0.9700	C18—N2	1.307 (3)
C8—H8B	0.9700	C18—H18	0.9300
C9—N1	1.469 (2)	C19—N2	1.449 (4)
C9—C10	1.506 (3)	C19—H19A	0.9600
C9—H9A	0.9700	C19—H19B	0.9600
C9—H9B	0.9700	C19—H19C	0.9600
C10—C11	1.381 (3)	C20—N2	1.442 (3)
C10—C15	1.391 (3)	C20—H20A	0.9600
C11—C12	1.384 (3)	C20—H20B	0.9600
C11—H11	0.9300	C20—H20C	0.9600
C12—C13	1.376 (4)		
			
O1—C1—C6	118.2 (2)	C12—C13—C14	122.1 (2)
O1—C1—C2	121.92 (18)	C12—C13—H13	119.0
C6—C1—C2	119.9 (2)	C14—C13—H13	119.0
C3—C2—C1	118.04 (19)	C13—C14—C15	119.5 (2)
C3—C2—C8	120.45 (18)	C13—C14—H14	120.3
C1—C2—C8	121.25 (17)	C15—C14—H14	120.3
C4—C3—C2	123.0 (2)	O2—C15—C14	122.6 (2)
C4—C3—H3	118.5	O2—C15—C10	117.40 (18)
C2—C3—H3	118.5	C14—C15—C10	120.0 (2)
C5—C4—C3	117.3 (2)	C12—C16—H16A	109.5
C5—C4—C7	122.3 (2)	C12—C16—H16B	109.5
C3—C4—C7	120.4 (2)	H16A—C16—H16B	109.5
C6—C5—C4	121.3 (2)	C12—C16—H16C	109.5
C6—C5—H5	119.4	H16A—C16—H16C	109.5
C4—C5—H5	119.4	H16B—C16—H16C	109.5
C5—C6—C1	120.6 (2)	N1—C17—C17^i^	111.36 (19)
C5—C6—H6	119.7	N1—C17—H17A	109.4
C1—C6—H6	119.7	C17^i^—C17—H17A	109.4
C4—C7—H7A	109.5	N1—C17—H17B	109.4
C4—C7—H7B	109.5	C17^i^—C17—H17B	109.4
H7A—C7—H7B	109.5	H17A—C17—H17B	108.0
C4—C7—H7C	109.5	C17—N1—C9	111.94 (15)
H7A—C7—H7C	109.5	C17—N1—C8	110.94 (14)
H7B—C7—H7C	109.5	C9—N1—C8	109.95 (15)
N1—C8—C2	112.72 (16)	C1—O1—H1	109.5
N1—C8—H8A	109.0	C15—O2—H2	109.5
C2—C8—H8A	109.0	O3—C18—N2	126.2 (2)
N1—C8—H8B	109.0	O3—C18—H18	116.9
C2—C8—H8B	109.0	N2—C18—H18	116.9
H8A—C8—H8B	107.8	N2—C19—H19A	109.5
N1—C9—C10	112.49 (16)	N2—C19—H19B	109.5
N1—C9—H9A	109.1	H19A—C19—H19B	109.5
C10—C9—H9A	109.1	N2—C19—H19C	109.5
N1—C9—H9B	109.1	H19A—C19—H19C	109.5
C10—C9—H9B	109.1	H19B—C19—H19C	109.5
H9A—C9—H9B	107.8	N2—C20—H20A	109.5
C11—C10—C15	118.63 (19)	N2—C20—H20B	109.5
C11—C10—C9	122.36 (19)	H20A—C20—H20B	109.5
C15—C10—C9	119.00 (18)	N2—C20—H20C	109.5
C10—C11—C12	122.5 (2)	H20A—C20—H20C	109.5
C10—C11—H11	118.8	H20B—C20—H20C	109.5
C12—C11—H11	118.8	C18—N2—C20	121.7 (2)
C13—C12—C11	117.3 (2)	C18—N2—C19	119.4 (3)
C13—C12—C16	121.1 (2)	C20—N2—C19	118.8 (3)
C11—C12—C16	121.6 (2)		
			
O1—C1—C2—C3	179.97 (17)	C10—C11—C12—C13	0.7 (3)
C6—C1—C2—C3	−0.9 (3)	C10—C11—C12—C16	−177.9 (2)
O1—C1—C2—C8	−5.9 (3)	C11—C12—C13—C14	−1.0 (3)
C6—C1—C2—C8	173.23 (18)	C16—C12—C13—C14	177.5 (2)
C1—C2—C3—C4	1.1 (3)	C12—C13—C14—C15	0.2 (4)
C8—C2—C3—C4	−173.15 (18)	C13—C14—C15—O2	179.7 (2)
C2—C3—C4—C5	−0.2 (3)	C13—C14—C15—C10	0.9 (3)
C2—C3—C4—C7	177.9 (2)	C11—C10—C15—O2	179.93 (18)
C3—C4—C5—C6	−0.7 (3)	C9—C10—C15—O2	−0.9 (3)
C7—C4—C5—C6	−178.9 (2)	C11—C10—C15—C14	−1.2 (3)
C4—C5—C6—C1	0.9 (3)	C9—C10—C15—C14	177.91 (19)
O1—C1—C6—C5	179.13 (19)	C17^i^—C17—N1—C9	80.0 (3)
C2—C1—C6—C5	0.0 (3)	C17^i^—C17—N1—C8	−156.7 (2)
C3—C2—C8—N1	−144.07 (17)	C10—C9—N1—C17	−157.41 (16)
C1—C2—C8—N1	41.9 (2)	C10—C9—N1—C8	78.8 (2)
N1—C9—C10—C11	−109.0 (2)	C2—C8—N1—C17	73.2 (2)
N1—C9—C10—C15	71.9 (2)	C2—C8—N1—C9	−162.47 (15)
C15—C10—C11—C12	0.4 (3)	O3—C18—N2—C20	178.2 (2)
C9—C10—C11—C12	−178.70 (19)	O3—C18—N2—C19	−0.4 (4)

Symmetry codes: (i) −*x*+1, −*y*+1, −*z*+1.

### Hydrogen-bond geometry (Å, °)

**Table:**

*D*—H···*A*	*D*—H	H···*A*	*D*···*A*	*D*—H···*A*
O1—H1···N1	0.82	1.98	2.705 (2)	147
O2—H2···O3	0.82	1.87	2.690 (2)	177
C18—H18···O3^ii^	0.93	2.56	3.368 (3)	145

Symmetry codes: (ii) −*x*+1, *y*+1/2, −*z*+3/2.

## References

[bb1] Bruker (2000). *SMART* and *SAINT* Bruker AXS Inc., Madison, Wisconsin, USA.

[bb2] Burnett, M. N. & Johnson, C. K. (1996). *ORTEPIII* Report ORNL-6895. Oak Ridge National Laboratory, Tennessee, USA.

[bb3] Farrell, J. R., Niconchuk, J., Higham, C. S. & Bergeron, B. W. (2007). *Tetrahedron Lett.* **48**, 8034–8036.

[bb4] Farrugia, L. J. (1997). *J. Appl. Cryst.* **30**, 565.

[bb5] Higham, C. S., Dowling, D. P., Shaw, J. L. & Farrell, J. R. (2006). *Tetrahedron Lett.* **47**, 4419–4423

[bb6] Hou, G.-G., Ma, J.-P., Wang, L., Wang, P., Dong, Y.-B. & Huang, R.-Q. (2010). *CrystEngComm*, **12**, 4287–4303.

[bb7] Liu, X. L., Shang, X. M., Tang, T. & Cui, D. M. (2007). *Organometallics*, **26**, 2747–2757.

[bb8] Sheldrick, G. M. (2008). *Acta Cryst.* A**64**, 112–122. 10.1107/S0108767307043930 18156677

[bb9] Spek, A. L. (2009). *Acta Cryst.* D**65**, 148–155. 10.1107/S090744490804362X PMC2631630 19171970

[bb10] Tshuva, E. Y., Goldberg, I., Kol, M. & Goldschmidt, Z. (2000). *Inorg. Chem. Commun.* **3**, 611–614.

